# Poliomyelitis; Apropos of the Spread Southward of the Disease; Recent Researches; Fallacies in Diagnosis; Misconceptions about Treatment

**Published:** 1911-04

**Authors:** Tom A. Williams

**Affiliations:** M. B., C. M. (Edin.); Washington, D. C.; Member Corresp. Soc. de Neurol. de Paris; Member Corresp. Soc. de Psychol de Paris; Member Assoc. de la Soc. de Med. Ment Clin.; Neurologist to the Epiphany Free Dispensary


### POLIOMYELITIS: APROPOS OF THE SPREAD SOUTHWARD OF THE DISEASE; RECENT RESEARCHES; FALLACIES IN DIAGNOSIS; MISCONCEPTIONS ABOUT TREATMENT.

By Tom A. Williams, M. B., C. M. (Edin.), Washington, D. C.

*Member Corresp. Soc. de Neurol. de Paris; Member Corresp. Soc. de Psychol de Paris; Member Assoc. de la Soc. de Med. Ment Clin.; Neurologist to the Epiphany Free Dispensary.*

This disease, the unusual prevalence of which caused so much alarm in New York and England since 1907, and even before, has this year become epidemic as far South as Washington, and seems likely to extend still farther.

As investigations more extensive than any of those in the past have recently been conducted not only in this country but abroad, and as the writer finds much uncertainty as to the results of these among medical men by whom he has been consulted, it seems wise to present now a short statement of some important facts about the disease. In order to prevent its spread, the co-operation of the general fractitioner is imperative, as was long ago found out by the Scandanavian Government Research Bureau. In those countries upon application a diagnostician or a pathologist is sent to any reported case, and a concerted effort is made by these traveling specialists to instruct practitioners in the special technique which has been adopted for the research of data, both ante and post-mortem.

In New York, a joint committee of the Neurological and Poediatrical Societies attempted a statistical investigation of 2,000 (estimated) cases, of which 750 were analyzed. They concluded that the disease was infectious but not contagious, that it was more fatal to life than commonly supposed, and that complete recoveries without paralysis were not so unusual as had been believed. In many cases the disease behaved like other infections and the general symptoms were often as prominent as the paralysis. The brain stem might be involved, but there were very few cases of encephalitis. Early meningitis was, however, very frequent.

Only two cases occurred in negroes. The epidemic began in June, and was at its height in September, the country cases beginning later. One epidemic spread along a trolley line. Poor and rich alike were affected and the incidence seemed proportional to population. The communicability equalled that of cerebrospinal meningitis. Relative immunity seemed to follow an epidemic. In the Massachusetts epidemic, evidence of contagion presented in 17% of cases.

The diarrhea which many authors believed important was not prevalent (14%) constipation being as frequent.

There was respiratory catarrh in 84 cases, of which 45 showed sore throat. Only 21 cases showed gastro-enteritis within two weeks before onset. 42% of the 283 cases under two years were at the breasts this seems to eliminate food infection. Many cases showed no *prodromata.*

The more frequently mentioned symptoms were restlessness, irritability, apathy, pain in back and limbs; pyrexia was absent in only 61 cases. In 111 cases the fever lasted over a week—its usual duration being two or three days. Vomiting occurred in nearly 25%. Rigor occurred in 61.

Of the *nervous symptoms,* restlessness was the most frequent; it occurred in 369 cases; headache in 162 cases, delirium 62 cases, convulsions 51 cases, twitchings 8 cases, apathy occurred in 294 cases, stupor in 71 cases, rigidity of the neck was reported in 121 cases. This, of course, indicates meningeal irritation, and is clinically very hard to differentiate from cerebrospinal meningitis. Meningeal irriation is also indicated by the pain and tenderness. This occurred in nearly all the cases for a day or two, and in 50 per cent. was quite marked, and in some of the cases excruciating. It was most frequent in the lower limbs and next to the trunk. There was skin eruption in 81 cases. Papules were the commonest, and they often covered the whole body. The significance of this has yet to be explained, as in Norway only one case occurred.

The invasion of the nerve centres is usually simultaneous, and in more than a quarter of the cases was complete the first day, and in 90% before the third day. Although the paralysis is well-kwown to be a flacoid one, rigidity preceded the relaxation in 38 cases of the New York epidemic. This, again, is indicative of meningeal irritation. Not only could the muscles of the limbs be affected, but those of respiration, deglutition, speech, ocular movements and of the bladder and rectum.

*The pathological conclusions* of the New York Committee were as follows:

1. That in poliomyelitis acuta there were both interstitial and parenchymatous lesions, but that the interstitial were of fundamental importance and the latter secondary.

2. That the ganglion cells were affected only when in contact with the interstitial process.

3. That the interstitial process was dependent upon the vessels for its character and localization.

4. That the lesion, while generally most marked in the anterior horns, was not confined to that portion of the gray matter, and hence the word "anterior" should not be used to designate the condition.

5. That the white matter of the cord was the seat of inflammatory changes of minor importance.

6. That the spinal infiltration was the essential element in the disease, and might be the origin of the infective agent.

7. That the involvement of the medulla, pons and basal ganglia always occurred in the fatal cases, though clinical experience in the last epidemic had shown that such involvement did not mean a fatal issue necessarily.

8. That in striking contrast to the cord, the ganglion cells in the medulla, pons and basal ganglia, even when near infiltrated zones, escaped serious alteration.

9. That the brain cortex may show evidences of vascular irritation and sometimes infiltration.

10. That the edema which is present plays an important role in explaining the transitory nature of the symptoms in the nonfatal cases.

11. That the predominating role ascribed to the central artery by previous observers was unjustifiable.

12. That there was no evidence of thrombosis.

13. That apparently the infective agent may affect any part of the brain stem in its initial lesion.

14. That it could not be determined from a study of the pathological histology whether the infection had a hematogenous or lymphogenous origin.

15. That while the central nervous system was the seat of the principal lesion in poliomyelitis acuta, changes in the internal organs of the body pointed to a general infection.

16. That the acute inflammation of the lymph apparatus connected with the intestinal tract might indicate the path of entrance of the infective agent.

Much fear has been created by the newspaper accounts of this disease. In consequence of the alarmist's attitude adopted in these, many people have taken their children away from what they believe to be infected areas. This attitude is quite unjustifiable, for reports of cases in the country districts are reaching me in considerable numbers, so that a child is perhaps as likely to contract the disease at country or sea-side as in town, the number of cases being generally proportional to population. Until the method of contagion is discovered, we do not know what precautions to adopt, but it might be prudent to restrict contact with other children, more especially in such frequented places as moving picture shows, nickel and dime theatres, churches, camp-meetings, crowded sea-side boarding-houses, and, perhaps, public play-grounds for general principles tell us that crowding favors contagion. But these are channels of infection, and I do not advocate their adoption, or the closing of schools on account of the disease, the incidence of which is comparatively small and the mortality minimal.

Shaffer has recently suggested that the avenue of infection is by abrasions in the skin.

### Researches into Etiology at the Rockefeller Institute of Preventive Medicine.

In 1907, there was a severe epidemic of poliomyelitis anterior acuta along the sea-board of New England. As a result there were many deaths, and over a thousand cases are now permanently crippled. Dr. Flexner and his associates had access to a large number of cases, and made numerous attempts to transmit the disease to monkeys by the inoculation of the subarachnoid fluid obtained by lumbar puncture of patients at different stages of the disease. They failed, as they had previously done with the other laboratory animals. ut the non-transmissability of the disease by this means made it probable that no ordinary bacterium was responsible for it.

In 1909, there was a less extensive epidemic; it was more local, and there were only a few hundred cases. This time the Rockefeller Institute succeeded in obtaining material from the central nervous system from two patients who died. One of these children, a case of average severity, as regards paralysis, died on the seventh day, and from it they succeeded in obtaining one inch of the lumbar cord. From a severe case which died on the fourth day, they obtained the whole cord but in both cases the brain was refused.

From this material, Drs. Lewis and Flexner have now inoculated eleven series of monkeys, and the virus has in no way diminished in potency. The animals are susceptible whether the virus is introduced into the centra nervous system or by the subcutaneous, peritoneal or intravenous route. Injection down the trachea has not, however, yet produced the disease. The lesions are always in the same locality, that is cerebello-spinal ganglia and roots and the meninges covering them and rarely in the cerebrum The lesions tend to preponderate low down. Of the eighty-one cases, the legs were affected in forty, both legs in twenty, all four limbs in ten, the arm in thirty-one, and the neck alone in one case, although it was often implicated along with other regions. In only eight cases were the bulb and cortex affected. In these facial paralysis was the feature.

It is a blood vascular disease, apparently, not traveling by lymph channels, except that the glands in the neighborhood of the subcutaneous inoculation become inflamed; but they never do except in the neighborhood, and never at all after internal inoculation. Moreover, little virus is found in the spleen, liver or bone marrow. Even the blood is of low virulence, and that only early in the disease.

*Nature of Virus.*—It is very active, invisible, even by special illumination. It is not arrested by the finest filter. So far, they have not succeeded in staining it nor in cultivating it, unless a transmission and cloudiness in certain media may be regarded as a culture. At all events, these cultures do not transmit the disease, thus differing from the invisible organism of pleuro-pneumonia. The question arises whether it is a sapiophyte, for so far, no invisible one is known. May it not be a bacterium after all, for it resists thirty days at 2 C.? Drying for seven days over caustic potash does not diminish its virulence, nor does admixture with glycerine. It even resists 5% phenol numberous suspensions in water and long keeping do not render it inactive, but it is sensitive to heat, becoming inert at 150.

*Transmission.*—It shows many analogies with the ciolococcus intravelularis, and Flexner believes that both organisms enter and leave the body via the nares, the cribriform plate and the cerebro-spinal fluid. It will be recollected that he proved this with regard to the Veichselbaum coccus by introducing cultures into the vertebralis and later finding the organism in the monkey's nares, and he has now found that at one stage of poliomyelitis, the cerebro-spinal fluid does show virulence, and suggests this route as worth inquiry. In this connection, recent work of Levauiti at the Pasteur Institute shows that the saliva is infective while still in the gland and suggests further investigation.

Flexner, however, from the character of the lesions, thinks that poliomyelitis begins in the meninges.

*Immunity.*—Even after a very slight attack, an animal strongly resists re-inoculation. This is only in conformity with are rarety of second attacks in human beings, although there have been a few cases where, as far as clinical evidence goes, what appears to be a poliomyelitis has occurred in persons already showing infantile paralysis. Relapses or recrudescences are perhaps less rare. Whether the strictly abortive attack confers immunity is another question which Flexner hopes will soon be decided by his experiments. It can be answered both by inoculation experiments and by the results of the immunity reactions. The material of this kind is still insufficient to permit report; but very soon there promises to be definite information; for several of the monkeys have manifested merely a slight general malaise without paralysis or even weakness. Those inexperienced in observing the animals might, indeed, have believed them quite in good health, as no fever is shown in the prodromal stage. But Flexner is unable to indicate as yet the clinical symptoms which precede the paralysis.

*Incubation* has varied from four to thirty-three days. The average is about eight days, as in human beings. The recovery of function, as in human beings, is complete after a slight attack, but only partial when the attack is severe.

*The mortality* seems to be higher than in human beings, 50% of the monkeys having died. As far as the as yet imperfect study of the symptoms permits, Flexner believes that Landry's ascending paralysis may be often due to this virus, a further observation will perhaps demonstrate.

It is interesting to note that although only monkeys are susceptible to this particular virus, other animals are attacked by a poliomyelitis, for instance, nervous distemper in a dog is of this nature, but pathologists have not succeeded in transmitting it. The poliomyelitis of chickens has not been successfully inoculated. In rabbits, the Shiga bacillus causes a hemorrhagic poliomyelitis, generally cervical. So it is quite clear that there is a whole genus of organisms capable of producing this anterior horn syndrome, but it is clear, too, from Flexner's researches that the human form is specific.

*Pathology.*—The post-mortem examination of the monkeys make it quite clear what clinicians and pathologists have for some time suspected that the affection is not confined to the anterior horns. In the monkeys which died, inflammation was always found in the intervertebral ganglia and spinal roots. The infection seems to seat in the pia, and to spread thence via the trabeculae into the central nervous system. Sections show a considerable small round cell infiltration of the gangia on the spinal roots. The cells are of mononuclear type and occur in the subrachnoid space, too, and in the cord, especially round the anterior horn, an abundant round-cell exudate and dilitation, enlargement and sometimes rupture of the blood vessels. This infiltration of the roots and ganglia explains the severe pains of which adult cases often complain. In the child, of course, the general distress often masks the local pain.

It is interesting to note that in Weichselbaum's laboratory, they have also succeeded in inoculating the monkey with the virus of anterior poliomyelitis.

We may have strong hope that in a very short time the completion of these researches will provide us with the means of immunizing human beings against still another contagious disease, or at least of cutting short an attack, as is now the case in cerebro-spinal meningitis. But even if this is not realized, Flexner's experiments will show us the path of infection, and thus furnish another means by which to prevent disease; for up till now, we have had to fight quite in the dark against an unknown foe, and means of prevention cannot but lack in precision under such conditions. But when we know the avenue of infection, means of prevention are often found to be simple. Even in the case of poliomyelitis, where we scarcely know the child is sick until the disease is at its worst, and paralysis has occurred a way may now be found.

*Diagnosis.*—The prominent features at the commencement of simple cases, are the flaccidity of the affected limb, and loss of the reflexes in which the affected muscles are concerned. But there are pitfalls for the general practitioner in the exporation of these latter. I find it not unusual, especially in children, that the reflexes are reported to be absent, when in reality the patient has suppressed the reaction by a rigid contraction of all the muscles of the limb being explored. No observation is of value unless the limb is placed in the correct position and all muscles are relaxed. A case of meningitis may in this way be mistaken for one of infantile paralysis, an error of diagnosis which might cost the patient's life.

Again, the transcendantly important *plantar response* is often misinterpreted. When the sole of the foot is tickled or scratched, the normal response often includes a rapid drawing up of the leg by flexion at hip, knee and ankle (dorsiflexion), and sometimes at toe (dorsally), as well, although the latter reaction is usually flexion, the extension which sometimes occurs being in part volitional. Now, this is not Babinski's sign, although it is often so called. To elicit this, a careful technique must be followed: all the muscles must be relaxed, the knee, hip and ankle passively semi-flexed, and it is best to gently hold the waist of the foot. With a brass pin in the other hand the sole of the foot should be firmly and slowly stroked from the heel towards the toe, first on the fibular side, and if no response occurs, then on the tibial side. If the toes do not yet move, the first stroke must be repeated and continued into a broad and sweeping mesial-ward movement across the pad at the base of the toes.

Babinski's sign consists of a slow extension of the great toe, after one of these stimuli *under these conditions.*

Accessory and important signs described by the same observer are the spreading of the four outer toes (the fan sign), and the sharp contraction of the tensor fasciae femoris muscle.

Extension of the toe sometimes recurs in meningitis and hence its erroneous declaration may cause a serious error of diagnosis.

Difficulties of diagnosis are presented also by (1) Keronig's sign, unusual distribution of paralysis, marked mental symptoms without paralysis, bulbar paralysis, gradual ascending or descending paralysis, resembling that described by Landry, cases with encephalitis. Examination by a skilled neurologist should be sought in such difficult cases.

A condition which might be mistaken for poliomyelitis is respiration slowed to a threatening of failure.

The investigation of these epidemics show that we must discard many old views about the early symptoms. The unsatisfactory histories and sometimes examination of dispensary cases, Dr. Emmet Holt believes, should prevent us from concluding that abortive cases are rare, and he believes that many of them are overlooked or miscalled. He believes, too, that a number of deaths from acute poliomyelitis are attributed in such cases as pneumonia, cerebro-spinal meningitis, encephalitis and Landry's paralysis. Gowers, too, has insisted upon the frequency of errors of diagnosis.

On the other hand, Dr. Lovett believes that during epidemics many other diseases are called poliomyelitis.

I cite as an instance of a difficulty which any neurologist will appreciate, a case seen with Dr. Copeland during this epidemic.

After one week of malaise and constipation, a girl of four years developed what was believed to be a sore throat, the neck being held stiff. Two days later, Dr. Copeland first saw her and found clear rigidity of neck, light Kernig's sign, a scaphoid abdomen and exaggerated patellar reflexes. The child was apathetic.

These signs of meningeal irritation created a suspicion of an invasion by the diplo-coccus intra-cellularis, especially as during the next four days the apathy increased to the point of unconsciousness, swallowing became more and more difficult, and the the rare one of syphilitic pseudoparalysis.

There also developed a large intention-tremor of the right arm and leg, and the power of articulation disappeared.

The dysphegia led to removal to a hospital so that skillful feeding could be secured. Dr. Copeland did not ascertain whether or not it was spasm or paralysis of the pharyngeal or other muscles which prevented swallowing, but the history and other symptoms point to paralysis as the likelier cause.

During this time the patellar reflexes disappeared and poliomyelitis was suspected.

The limbs were no longer rigid, nor was there distinct flaccidity. The plantar response had not been that of Babinski.

It was two weeks after onset when I saw the child. She then showed a slight concomitant strabismus and distinct facial asymmetry, the folds upon the right side being much more distinct, but there was no apparent diminution of facial movements. The neck was turned to the left, and appeared to be more able to turn in that direction against resistance than in the contrary direction.

I could detect no inequality of power in the movements of the hands and arms, neither being used very strongly, shyness or apathy may explain this. The extension of the right leg upon the thigh was distinctly weaker than that of the left side. I could detect no inequality in other movements, but none were vigorous, for the strong child she seemed.

In walking, however, progress was dysergic, more especially in the right leg, the base was widened by spreading the legs, and she tended to utilize the support of the furniture when within reach.

The reflexes were all brisk, but the force with which the right leg extended on tapping the patellar ligament was much less than that of the left. The abdominal reflexes were present as were the plantar, but the reflex of the right foot was less complete than that of its fellow, and a wide spreading of the outer toes when the sole was stroked (the fan sign), gave clear evidence of a slight impairment of the function of the pyramidal fibres somewhere in their course.

Babinski's combined flexion sign was seen at the right groin when the child rose up to sitting posture, and when she lifted the leg from the bed, the contralateral pressure on the right heel was less than when the left one pressed on the bed.

The mother reports that the child has lost her cheerfulness and is both apathetic and querulous.

I explain these symptoms as the result of an invasion by the causative virus of poliomyelitis affecting mainly the region of medulla oblongata and pons varolii.

*The Course and Treatment.*—Wickman reports 10% mortality in the Scandanavian epidemic. In the New York epidemic, about 6% recovered completely, while in 86% some paralysis remained. The deaths of reported cases were infrequent, but it is likely that many cases died without diagnosis. During the active stage, rest and light diet are the most important needs. Drugs are not indicated, and the general treatment of fever should be followed.

The indications for treatment of the paralyzed muscles are:

(1.) To aid the rapid absorption of the inflammatory exudate in and around the central nervous system. Potassium iodide is generally recommended for its deobstruent effect. It is possible that some more active fibrolytic might here find a use, but I am not aware of its trial.

(2.) To favor regeneration of the nerve elements by good nutrition.

(3.) Most important is it to maintain the life of the muscles of which the function and nutrition are interfered with by destruction of their neurones in the anterior horn of the spinal cord, including their axonal prolongation into the peripheral motor nerve. There is much misapprehension concerning the means of doing this. Vibration, massage, passive movements and hydrotherapy are quite secondary in importance to direct stimulation of muscle contractility by *galvanic electricity.* This is the only means which will keep alive the myotomes during the course of regeneration of their nerves. As those of which the cells are entirely destroyed will never regenerate, it is impossible, at first, to predict the amount of voluntary muscle which will remain to the patient, but it is our duty to keep alive the whole muscle in the hope that when exudate and edema subside, there will remain sufficient innervation to give useful movements.

Degeneration of nerve is well within three days, and at the end of ten days is decided. Regeneration is a matter of months. Hence, electrical stimulation should be begun early, contrary to a very general notion, which has confused the contra-indications of irritative conditions of the nerves with the indications just outlined. Galvanism to the muscles can have no possible irritating effect upon the inflammatory area in the spinal cord, and if it should be begun immediately the fever subsides and the patient's general state is such as to permit of its employment without causing perturbation of the mind.

(4.) Preventions and correction of deformation.

It is most important from the very beginning to prevent contracture and malposition. Were this done much orthopedic surgery could be dispensed with.

*Preventive Inoculation.*—Efforts at preventive and curative inoculation are being made in the laboratories of the Pasteur Institute, of the Rockefeller Institute, and in the laboratory of Wiechelbaum. The results of Leviditi are so far the most promising. He, too, has discovered what resembles an organism by centrifugalizing and diluting the precipitate, which it then stained by Loffler's method. It occurs in large numbers as an extremely small, round, or slightly oval diplo or staphylococcus. With the Giensa method, what appeared to be nuclei are shown in blue. These bodies differ from those found in control cultures.

The preventive inoculation used by Leviditi is performed with the spinal-cord of infected animals. This substance is viruent when intracerebrally injecting. Heating to 56 destroys the vaccine.

Hexamethylamine, by the mouth, is now given by Dr. Starr as a routine, on account of the fact that it is excreted into the cerebro-spinal sac.

The New York committee lay great stress upon the prolonged and frequent hot bath in the treatment, not only of the acute and painful stage, but during the time when feeble attempts are being made by the little patient to move his paralyzed muscles. The bath should be at least twelve inches deep, the temperature 100 F., gradually increased to 104 F. At least fifteen minutes should be spent in it three or four times a day, and the bath should not be permitted to cool. After the bath, the child should be dried in bed while wrapped in a blanket. The Committee has no doubt that the baths both promote sleep and relieve pain by preventing tension upon the muscles and joints. Not only so, but it is much less painful to straighten flexed limbs while they are warm and suspended in the bath. Again, the perfectly equal support of the limbs while floating in water makes possible voluntary movements which cannot be performed while the weight of the limb has to be overcome. In a child, it is particularly difficult to excite voluntary effort against drag or discomfort. A systematic method of exercises while in the bath must be devised, and these must be made into a game, so that the child's interest may be enlisted. Each case will require different exercises, in accordance with the incidence of the paralysis.

The psychic factor is of the greatest importance in this part of the management of the case. Later on, too, one must not neglect the reticence and tendency to diffidence which the disability may foster in the child. On the other hand, exaction and selfishness must be guarded against. Stories of great men who have overcome physical infirmities are of much help. A small country town is, perhaps, the best environment for such people, whose range of occupation is not so limited as one at first supposes. If it is necessary to turn to a purely intellectual pursuit, tuberculosis must be obviated by specially devised exercises and as much open air as possible; cramped positions must be avoided.

Locomotion is often possible by means of a rolling platform, modified roller skates, or something of the kind, for every encouragement must be given to the use of each shred of muscle tissue spared.

Besides the spychic factor a great obstacle to muscle contraction is stretching of the muscle fibres by contracture of the antagonists from the permitting of incorrect attitudes. These can be overcome in the first stages in the bath, and later on by gentle massage, bandaging and apparatus.

My experience shows, contrary to the belief of this committee, that *galvanic electricity,* so far from irritating and increasing pain, actually *soothes and relieves pain,* so that after its application, an extended position, which before was intolerable, is for a while comparatively easy of achievement. This apparent discrepancy of experience is perhaps due to the great imprecision with which have been reported to the New York Committee the manner of application of electrical treatment and the period of the disease at which it has been applied.

For the detailed account of some exercises which they have employed, and for the further details as to pathology, epidemiology, etc., of poliomyelitis as revealed by recent researches, I must refer the reader to the report of the New York Committee, published as No. 6 of the Nervous and Mental Monograph Series, the monograph of Wickman (Berlin, 1907), the recent publications of the Rockefeller Institute for Preventive Medicine, and those of Lividiti, in the transactions of the Biological Society of Paris.

I need not go into detail to refute the exaggerated claims made by the newspapers concerning a special form of massage employed in New Jersey, for it has no special validity beyond the glorification of those concerned in the eyes of the ill-informed.

